# Idiopathic splenic infarcts in a patient with situs inversus totalis: a case report

**DOI:** 10.1093/jscr/rjae605

**Published:** 2024-10-17

**Authors:** Bashar Abunasser, Hisham Issa Shabani

**Affiliations:** Department of Surgery, Specialty Hospital, Jaber Ibn Hayyan St., Shmeisani, Amman 11193, Jordan; Department of Surgery, Specialty Hospital, Jaber Ibn Hayyan St., Shmeisani, Amman 11193, Jordan

**Keywords:** splenic infarction, situs inversus, situs inversus totalis, case report

## Abstract

Situs inversus totalis is a rare congenital abnormality characterized by a mirror-image transposition of both the abdominal and the thoracic organs. Splenic infarctions are considered a rare cause of abdominal pain, although the exact prevalence is unclear. We present a rare case of a 62-year-old male who presented to the emergency department with abdominal pain and was found to have large splenic infarcts with situs inversus totalis on computed tomography. The patient was admitted and treated conservatively. In conclusion, diagnosing situs inversus in cases of emergency is important because patients will present with abnormally located symptoms, and splenic infarction should be kept in mind when considering the differential diagnosis of abdominal pain despite its rarity.

## Introduction

Situs solitus describes the normal anatomy, situs inversus is the complete reversal, and situs ambiguous is used for any other abnormality of left–right development. Frequency of situs inversus is 1:10 000 and is more frequent in males: 1.5:1. Advanced imaging modalities can be used to assess fine anatomical details, which play a crucial role in these cases to plan radiologic or surgical interventions [1].

Splenic infarction occurs when blood flow to the spleen is compromised, causing tissue ischemia and eventual necrosis. Splenic infarction may be the result of arterial or venous occlusion. Occlusion is usually caused by bland or septic emboli as well as venous congestion by abnormal cells. Infarction may involve a small segmental area of the spleen or may be global, depending on which vessel is occluded. This occurrence is caused by a wide variety of underlying disease states with a prognosis dependent on the causative illness [2].

Causes of splenic infarction include thromboembolic disease, hematologic diseases, hypercoagulable states, blunt abdominal trauma, and pancreatic disorders [2].

Treatment of splenic infarction ranges from supportive care to splenectomy.

## Case presentation

A 62-year-old male, known to have situs inversus totalis with a history of coronary artery bypass surgery, presented to the emergency department complaining of generalized abdominal pain of 1 week duration that was responding to simple analgesia, but it became severe in the past 2 days. The pain radiates to the right flank and is associated with anorexia and one episode of vomiting 3 days prior to presentation. His last bowel motion was 1 day prior to presentation, and he has been having hard stools during this week. He did not complain of fever, chills, or urinary symptoms. He did not have any history of trauma recently.

On examination, the patient had a distended abdomen with epigastric, right upper quadrant, and right flank tenderness. He had diminished bowel sounds. His heart sounds were normal (dextrocardia) with clear lung fields on auscultation.

## Investigations

Initial lab investigations revealed an elevated WBC count of 13.2 × 10^9^/L (neutrophils: 77%). His hemoglobin was 15.2 g/dl, and his platelets count was 300 × 10^9^/L.

His serum urea, creatinine, electrolytes, liver function tests, amylase and lipase, PT/INR, and PTT were all within normal limits.

**Figure 1:**
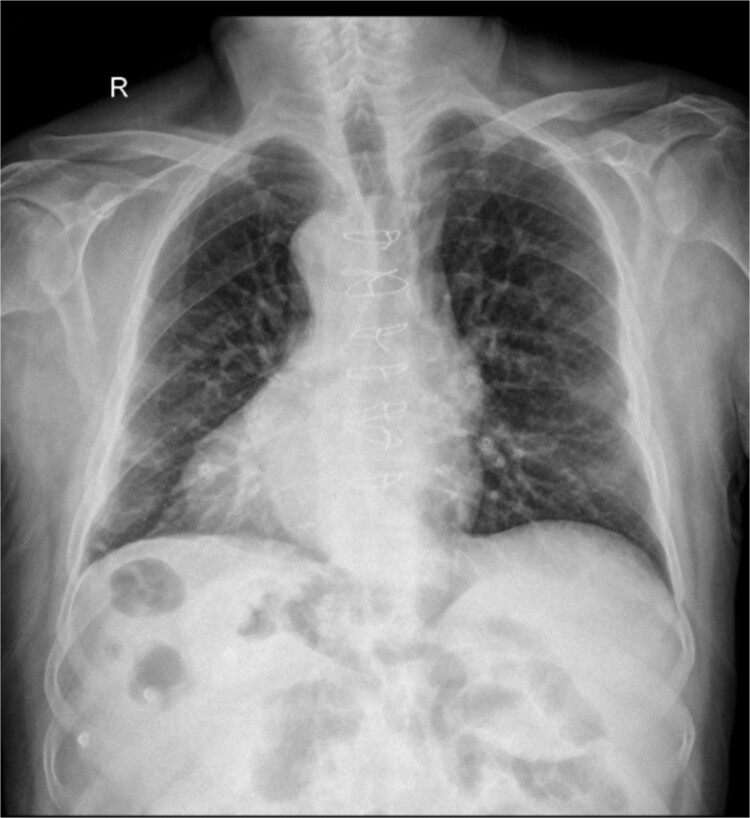
Chest X-ray showing dextrocardia.

A chest X-ray showed dextrocardia as seen in Fig. 1.

**Figure 2:**
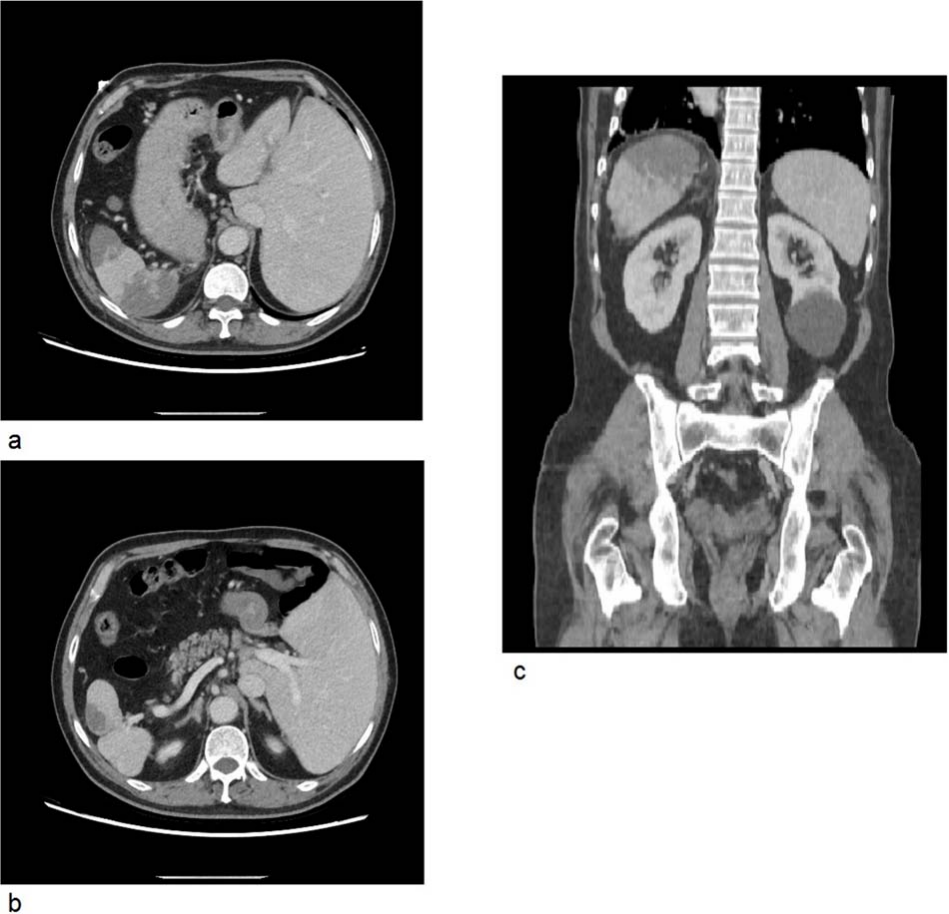
(a) CT axial cross section showing the liver on the left side of the abdomen and the spleen on right side with multiple splenic infarcts. (b) CT axial cross section showing patent splenic vein. (c) CT coronal cross section showing splenic infarcts as well as a left kidney cyst.

A computed tomography scan of the abdomen and pelvis with IV contrast was done (Fig. 2); situs inversus totalis was noted, large splenic infarcts were seen, and the superior mesenteric vein, splenic vein, and portal vein were patent. A 7.5 cm cyst was seen in the left kidney.

## Course in hospital

The patient was admitted for supportive care with IV fluids, analgesia, and anticoagulation.

He was given a bolus of IV fluids (normal saline 0.9%). For analgesia, he was given paracetamol four times daily and morphine PRN. He was started on therapeutic enoxaparin 1 mg/kg twice daily.

He was evaluated by cardiology; his ECG showed a normal sinus rhythm and Troponin-I was negative. An echocardiogram was done; it did not show any structural abnormalities and was unremarkable.

He was also evaluated by hematology; a thrombophilia panel (Protein S, Anti-thrombin assay, Anticardiolipin Ab, Antiphospholipid Ab, Factor V Leiden, Factor II, MTHFR gene) was done, and it was normal.

The patient was doing well on supportive management; his pain improved; he was tolerating diet and passing bowel motions. He was discharged home on the second day of admission on therapeutic Enoxaparin.

## Discussion

Situs inversus totalis is a rare congenital abnormality characterized by a mirror-image transposition of both the abdominal and the thoracic organs. This is a global defect of situs orientation, as the failure to generate normal left–right asymmetry results in a spectrum of laterality disturbances. This condition might cause difficulties during diagnostic and therapeutic procedures [1].

Syndromes associated with abnormal situs include polysplenia syndrome, asplenia, or Ivemark’s syndrome [1]. Polysplenia is seen in 20% of cases of SI and belongs to its own group of heterotaxic syndromes [3]. There are a few case reports of patients with splenic infarcts or splenic rupture with situs inversus and polysplenia, and some of them needed surgical intervention [3, 4]. However, our patient had a single normal-sized spleen in the right upper quadrant.

Diagnosing situs inversus in cases of emergency is important because patients will present with abnormally located symptoms, and in these cases, imaging studies are helpful in clarifying the diagnosis and aiding to plan the management plan.

Ultrasound and CT scans can be sufficient to diagnose splenic infarctions. However, in cases of situs inversus, additional investigations may be required to identify the causes and possible associated anomalies [4].

This is a single case of a patient with two uncommon diagnoses; we recommend these cases to be followed up by a multidisciplinary team because joining efforts from different professionals would improve patient management.

## Conclusion

In conclusion, diagnosing situs inversus in cases of emergency is important because patients will present with abnormally located symptoms, and splenic infarction should be kept in mind when considering the differential diagnosis of abdominal pain despite its rarity.
